# Phenotypic, morphological, and metabolic characterization of vascular‐spheres from human vascular mesenchymal stem cells

**DOI:** 10.1002/jemt.23918

**Published:** 2021-08-27

**Authors:** Sabrina Valente, Carmen Ciavarella, Anna Hernández‐Aguilera, Fernández‐Arroyo Salvador, Marina Buzzi, Jorge Joven, Gianandrea Pasquinelli

**Affiliations:** ^1^ DIMES – Department of Experimental, Diagnostic and Specialty Medicine University of Bologna Bologna Italy; ^2^ Unitat de Recerca Biomèdica Hospital Universitari de Sant Joan, IISPV, Universitat Rovira i Virgili Reus Spain; ^3^ Campus of International Excellence Southern Catalonia Tarragona Spain; ^4^ Emilia Romagna Cord Blood Bank – Transfusion Service IRCCS Azienda Ospedaliero‐Universitaria di Bologna Bologna Italy; ^5^ Subcellular Nephro‐Vascular Diagnostic Program, Pathology Unit IRCCS Azienda Ospedaliero‐Universitaria di Bologna Bologna Italy

**Keywords:** arteries, human mesenchymal stem cells, spheres, stem cell metabolomics

## Abstract

The ability to form spheroids under non‐adherent conditions is a well‐known property of human mesenchymal stem cells (hMSCs), in addition to stemness and multilineage differentiation features. In the present study, we tested the ability of hMSCs isolated from the vascular wall (hVW‐MSCs) to grow as spheres, and provide a characterization of this 3D model. hVW‐MSCs were isolated from femoral arteries through enzymatic digestion. Spheres were obtained using ultra‐low attachment and hanging drop methods. Immunophenotype and pluripotent genes (SOX‐2, OCT‐4, NANOG) were analyzed by immunocytochemistry and real‐time PCR, respectively. Spheres histological and ultrastructural architecture were examined. Cell viability and proliferative capacity were measured using LIVE/DEATH assay and ki‐67 proliferation marker. Metabolomic profile was obtained with liquid chromatography–mass spectrometry. In 2D, hVW‐MSCs were spindle‐shaped, expressed mesenchymal antigens, and displayed mesengenic potential. 3D cultures of hVW‐MSCs were CD44^+^, CD105^low^, CD90^low^, exhibited a low propensity to enter the cell cycle as indicated by low percentage of ki‐67 expression and accumulated intermediate metabolites pointing to slowed metabolism. The 3D model of hVW‐MSCs exhibits stemness, dormancy and slow metabolism, typically observed in stem cell niches. This culture strategy can represent an accurate model to investigate hMSCs features for future clinical applications in the vascular field.

## Graphical abstract

hVW‐MSCs are able to form spheres, characterized by high NANOG and CD44 expression, low ki‐67 levels and slow metabolism. hVW‐MSC spheres represent an in vitro model of stem cell niche, suitable for investigating MSCs in the vascular field.

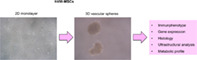

## 1 INTRODUCTION

Human mesenchymal stem cells (hMSCs) are multipotent fibroblast‐like cells, residing in human tissues including fresh and cadaveric arteries (Pasquinelli et al., 2010; Valente et al., 2014) and endowed of specific skills such as self‐renewal and multilineage plasticity. In vivo, they live in a three‐dimensional (3D) hypoxic microenvironment identified in multiple adult organs and tissues (Li & Xie, 2005) as a reservoir of stem cells in an undifferentiated and quiescent state. In vitro, hMSCs are cultured in a two‐dimensional (2D) plastic adherent monolayer according to International Society for Cellular Therapy (ISCT) minimal criteria (Dominici et al., 2006). MSCs are promising candidates in the regenerative medicine field, and hence they are characterized by multilineage differentiation, migration, immunosuppressive and anti‐inflammatory properties (Baer et al., 2010; Baraniak & McDevitt, 2010). The 3D culture strategy resembles the physiological microenvironment of the native niche by keeping the cell–cell interactions (Page, Flood, & Reynaud, 2013) and preserving the stemness features (Cesarz & Tamama, 2016).

Spheroids, so called for their spherical shape, are 3D symmetric cellular aggregates floating in the culture medium. Multiple approaches have been developed to generate spheroids based on cellular adhesiveness promotion and preventing contact with the substrate (Foty, 2011; Liu et al., 2013; Tsai, Liu, Yuan, & Ma, 2015; Wu, Di Carlo, & Lee, 2008; Yuhas, Li, Martinez, & Ladman, 1977; Zimmermann & McDevitt, 2014), including: spinner flasks using constant agitation; liquid overlay technique employing plastic surface precoated with organic matrix; hanging drops exploiting the gravity; non‐adherent surfaces such as plastic ultra‐low attachment culture substrates; microfluidic system applying a continuous perfusion; more recently, chitosan biomaterials embedding in gel.

Spheroids have been widely used for studying tumor biology, for recreating 3D native pseudotissues in tissue engineering (Fennema, Rivron, Rouwkema, van Blitterswijk, & de Boer, 2013) and in embryology as embryoid bodies for studying the embryogenesis and organogenesis (Mueller‐Klieser, 1997).

Spheroids may be transplanted and enhance bone and cartilage regeneration as well as they may improve the wound healing, angiogenesis and cardiac functions (Sart, Tsai, Li, & Ma, 2014). Recent studies showed an enhanced therapeutic potential, clonogenicity (Guo, Zhou, Wang, & Wu, 2014), multipotent differentiation (Wang et al., 2009), cell survival, angiogenic potential (Bhang, Lee, Shin, Lee, & Kim, 2012) and anti‐inflammatory properties (Bartosh et al., 2010) in hMSC spheres. These studies address mainly the beneficial properties of spheres, whereas a broad characterization of hMSC under 3D culture conditions remains largely incomplete. In this study, we characterized hVW‐MSCs‐derived spheres, including immunophenotypic and molecular profile, morphology, histological and ultrastructural investigation, proliferative capacity and metabolomic profile. We demonstrate that hVW‐MSC spheres gain a dormant proliferative and metabolic status that hypothetically approximates the native niche progenitors.

## 2 MATERIALS AND METHODS

### 2.1 Isolation and characterization of human vascular wall—mesenchymal stem cells (hVW‐MSCs)

The study was conducted on cryopreserved healthy human femoral arteries destined to disposal, provided by the Cardiovascular Tissue and Cord Blood Bank, University Hospital S. Orsola—Malpighi (Bologna, Italy), in accordance with the Local Ethics Committee Approval (APP‐13‐01). Tissues were digested with 0.3 mg/mL Liberase type II (Liberase TM Researche Grade, Roche) in serum‐free Dulbecco's modified Eagle's medium (DMEM; Sigma–Aldrich) with 1% antibiotics (Sigma–Aldrich) at 37°C in a rotor apparatus overnight (on), then filtered through cell strainers (100–70–40 μm) and centrifuged at 1200 rpm (Valente et al., 2014). Final cell suspensions were cultured in DMEM with 10% Fetal Bovine Serum (FBS; Sigma–Aldrich) and expanded in‐vitro, in incubator at 37°C/5% CO_2_/95% humidity. hVW‐MSCs at early passages (P2‐P3) were used for all the experiments.

### 2.2 Immunophenotype characterization

For flow cytometry, hVW‐MSCc were detached, counted and incubated with 1 μg antibody/10^6^ cells of unconjugated CD44, CD90, CD105, CD166, CD146, Platelet derived growth factor receptor‐β (PDGFR‐β), Neuron glial antigen 2 (NG2), CD133 and CD34, and conjugate (CD45) antibodies according to datasheet's instructions. Cells were washed with PBS and labeled with appropriate fluorescent probes in the dark, rinsed again before analysis in a flow cytometer (FACSAria, Becton Dickinson); the negative control was labeled with secondary antibody only; about 10,000 events were collected and data were elaborated using the FACSDiva Software (Becton Dickinson). Each antibody was diluted in 1% bovine serum albumin (BSA) in PBS and incubated for 40 min at 4°C. In addition, 6 × 10^5^ hVW‐MSCs were seeded on glass, fixed in 2% paraformaldehyde for 4 min at room temperature (rt). For nuclear or cytoplasmic antigens, cells were permeabilized with 1% Tryton X‐100 in PBS for 4 min at rt. Cells were blocked with 1% BSA for 30 min at rt and stained with ki‐67, Notch‐1 and Runx‐1 antibodies for 1 hr at 37°C in a wet chamber. After washing, samples were stained with AlexaFluor‐488 or AlexaFluor‐546 secondary antibodies in the dark, counterstained with Pro Long anti‐fade reagent with DAPI (Molecular Probes, Milano, Italy). Antibodies and respective dilutions are reported in Table 1. Negative controls were performed omitting the primary antibody. Samples were observed in a Leica DMI6000 B inverted fluorescence microscope (Leica Micro‐systems; Wetzlar, Germany).

**TABLE 1:** List of antibodies used for immunophenotypic analysis

Antibody	Dilution	Manufacturer
CD44	1:100	BD Biosciences Pharmingen
CD90	1:100	BD Biosciences Pharmingen
CD105	1:100	BD Biosciences Pharmingen
CD166	2 μL/10^6^ cells	BD Biosciences Pharmingen
Ki‐67	1:100	Novocastra
Runx‐1	1:250	Epitomics
α‐SMA	1:9,000	Sigma–Aldrich
PDGF‐Rβ	1:200	R&D Systems
NG2	1:50	R&D Systems
CD34	1:80	Dako
CD133	10 μL/10^6^ cells	MACS Miltenyi Biotech
CD45	10 μL/assay	ACZON
CD146	1:80	Abcam
Cx‐43	1:50	Invitrogen
Notch‐1	1:50	Santa Cruz Biotechnology
Alexa Fluor 488	1:250	Invitrogen
Alexa Fluor 546	1:250	Invitrogen

Abbreviations: α‐SMA, alpha smooth muscle actin; CD, cluster of differentiation; Cx‐43, Connexin‐43; NG2, neural/glial antigen 2; Notch‐1, notch homolog translocation associated 1; PDGF‐Rβ, platelet derived growth factor beta; Runx‐1, runt‐related transcription factor‐1.

### 2.3 Multilineage differentiation assays

For adipogenic and osteogenic assays hVW‐MSCs were plated at the density of 20 × 10^4^/well and 10 × 10^4^/well in 12‐well plates, respectively, and grown with StemPro Adipogenesis and Osteogenic Differentiation Medium (Life Technologies); cells cultured in DMEM 10% FBS were used as controls. After 14 days, adipogenic‐induced cells were formalin‐fixed and stained with Oil Red O for analysis of the cytoplasmic lipidic droplets. After 21‐days, osteogenic‐induced cells were formalin‐fixed and stained with Alizarin Red for calcium deposit evaluation.

For chondrogenic assay, 2.5 × 10^5^ hVW‐MSCs were pelleted in polypropylene conical tubes chondrogenic differentiation medium (StemPro Chondrogenic Differentiation Kit; Life Technologies), according to the manufacturer's instructions. Control cells were cultured in the same way in DMEM 10% FBS. After 7 days, cells were formalin fixed, paraffin embedded and stained with Alcian Blue.

### 2.4 hVW‐MSCs spheroids: cell number composition, size and growth curve

To generate spheroids, hVW‐MSCs were detached, pelleted, counted and filtered through a 40 μm cell strainer before plating in ultra‐low attachment 6‐well plates at the density of 1 × 10^5^ cells/well or establishing hanging drops of 35 μL with 2 × 10^4^ cells each drop both cultured in DMEM 10% FBS. After 3 days, spheroid formation was observed and pictured with the inverted light microscope (LM) before the collection for morphological, immunophenotypic and metabolomic analysis. To calculate the cell composition of individual spheroid, they were mechanically and enzymatically dissociated with trypsin in single cells and seeded on glass cover slips to allow cell adhesion. After washing with PBS, the samples were methanol fixed for 10 min, rinsed again and stained with crystal violet dye for 30 min at RT. Each sample was manually counted under a microscope equipped with an ocular micrometer and using a hemocytometer. Other spheroids (*n* = 5) were transferred in single ultralow attachment 24‐well plate and let floating in DMEM medium with 10% FBS for up 18 days capturing images every 3 days. For each sample, diameters were measured and expressed as average values.

### 2.5 hVW‐MSCs spheroids: immunophenotype examination

The immunophenotype of 3D cultures hVW‐MSCs was investigated through immunocytochemistry using a non‐biotin amplified method (NovoLink Polymer Detection Kit; Novocastra, Newcastle upon Tyne, UK) according to manufacturer's instructions. Spheres were formalin fixed and embedded in paraffin; 3 μm thick sections were cut, dewaxed in xylene and rehydrated in graded alcohols. The antigen retrieval was performed using citrate buffer at pH 6, at 120°C, 1 atm for 21 min. After endogenous peroxidase activity neutralization, specimens were labeled with a broad panel of antibodies against CD44, CD105, CD90, ki‐67, Runt related transcription factor‐1 (Runx‐1), Alpha—smooth muscle actin (α‐SMA), PDGFR‐β, Stromal cell surface marker‐1 (Stro‐1), CD34, Nestin, CD146 and Connexin‐43 (Cx‐43) overnight at 4°C in a wet chamber; all antibodies were diluted in 1% BSA in PBS as listed in Table 1. Further, the sections were exposed to 3,3′‐diaminobenzidine (DAB) substrate/chromogen, counterstained with hematoxylin, dehydrated, coverslipped, and viewed in a LM. Digital images were acquired at 10× magnification using Image‐Pro Plus 6 software (Media Cybernetics). To determinate the cycling cells and their position inside the spheroids, the ki‐67 intensely stained cells were manually counted and the values were expressed as absolute values.

### 2.6 Pluripotent gene expression

Total RNA was extracted from hVW‐MSCs spheres using TRIreagent, according to the manufacturer's indications (TRIzol reagent; Invitrogen). One μg of RNA was reverse transcribed in a 20 μL volume of reaction using a High Capacity Reverse Transcription Kit (Applied Biosystems, Carlsbad, CA). Real‐Time PCR analysis was performed using the SYBR green approach (Power Sybr Green PCR Master Mix) using specific couples of primers (Sigma–Aldrich; listed in Table 2) and carried out in an ABI Prism 7000 Sequence Detection System (Applied Biosystems). Each assay was run in triplicate and target gene expression was normalized to glyceraldehyde 3‐phosphate dehydrogenase (GAPDH). Relative quantification of mRNA expression was calculated with 2^−ΔΔCt^ method. Results were expressed as fold changes relative to hVW‐MSC grown in 2D condition.

**TABLE 2:** Primer sequences

Gene	Primer sequence
GAPDH	FWD 5′‐AATGGGCAGCCGTTAGGAAA‐3′ REV 5′‐AGGAGAAATCGGGCCAGCTA‐3′
SOX‐2	FWD 5′‐AGGATAAGTACACGCTGCCC‐3′ REV 5′‐TAACTGTCCATGCGCTGGTT‐3′
OCT‐4	FWD 5′‐TGAGTAGTCCCTTCGCAAGC‐3′ REV 5′‐GAGAAGGCGAAATCCGAAGC‐3′
NANOG	FWD 5′‐ACCTACCCCAGCCTTTACTC‐3′ REV 5′‐GGACTGGATGTTCTGGGTCT‐3′

Abbreviations: FWD, forward; GAPDH, glyceraldehyde 3‐phosphate dehydrogenase; OCT‐4, octamer‐binding transcription factor‐4; REV, reverse; SOX‐2, sex determining region Y‐box 2.

### 2.7 hVW‐MSCs spheroids: internal and external organization

For histology, hVW‐MSCs spheroids were formalin fixed, paraffin embedded, cut into 3 μm‐thick sections and stained with Hematoxylin and Eosin using a standard protocol. For Scanning Electron Microscopy (SEM), spheroids were plated on polylysine pretreated coverslips glass to facilitate the cell adhesion for 4 hr. After washing, the samples were fixed in 2.5% buffered glutaraldehyde (TAAB Laboratories, England, UK) o/n at 4°C, rinsed, post‐fixed in 1% osmium tetroxide in 0.1 M phosphate buffer for 1 hr, wet in distilled water and dehydrated with increasing concentration of ethanol (from 70 to 100%) at rt for 15 min each passage. To dry the samples, they were plunged in a 1:1 solution of absolute ethanol and hexamethyldisilazane (HMDS, Fluka Analytical, Sigma, Steinheim, Germany) followed by absolute HMDS for 30 min at rt and air‐dried in the last passage. The samples were attached on aluminum supports (Multilab type stub pin ½, Surrey, UK) using silver paste, coated with 10 nm thick layer of gold in a Balzers MED 010 sputtering device (Balzers Union FL 9496, Furstentum, Liechtenstein) and observed using a Philips 505 (FEI Company) Scanning Electron Microscope at 15 kV. For Transmission Electron Microscopy (TEM), spheroids were transferred in microtubes, washed in phosphate buffer and fixed in Karnowsky fixative (2% glutaraldehyde and 4% formaldehyde in 0.1 M phosphate buffer). After 1% osmium tetroxide post‐fixation and ethanol dehydration (from 30 to 100%), specimens were embedded in Araldite resin, sectioned, counterstained with uranyl acetate and lead citrate, and examined with a Philips CM10 (FEI Company, Milan, Italy) Transmission Electron Microscope equipped with a Gatan camera; digital images were captured using the FEI proprietary software Olympus SIS Megaview SSD digital camera.

### 2.8 hVW‐MSCs spheroids: viability and internal live/dead cell distribution

The distribution of viable cells inside the spheroids was investigated using the LIVE/DEAD® Viability/Cytotoxicity Assay Kit (Molecular probes, Invitrogen) performed in accordance to manufacturer's instructions. Calcein AM and ethidium homodimer (EthD‐1) probes were used to identify respectively the live cells (intense green signal) and dead cells (bright red fluorescence). The staining was analyzed in a Leica TCS SL Laser Scanning Confocal Microscope (Leica Micro‐systems; Wetzlar, Germany).

### 2.9 hVW‐MSCs spheroids: metabolomic activity

1 × 10^5^ hWV‐MSCs were cultured in basal medium with 10% FBS both in adhesion to plastic dish (2D) and 3D condition as vascular‐spheres, recovered and processed for metabolomics analysis. For 2D, the cells were cultured until confluence while vascular‐spheres were obtained after 3 days of culture in ultralow attachment 6‐well plates. To normalize the metabolomics data, the total cellular proteins of monolayer and spheroids were extracted using lysis buffer (KH_2_PO_4_ 0.1 M pH 7.5, NP‐40 1%, and 0.1 mM α‐glycerolphosphate, plus the complete protease inhibitors cocktail, Roche Diagnostics) and quantified in a spectrophotometer with the Bio‐Rad Protein Assay (Bio‐Rad Laboratories, Hempstead, UK). All experiments were carried out six times. Metabolomic analyses have been performed as described elsewhere (Riera‐Borrull et al., 2016). Metabolites investigated are listed in Table 3.

**TABLE 3:** List of metabolites investigated

Metabolite	hVW‐MSC monolayer (μmol/L·mg total protein)	Vascular‐sphere (μmol/L·mg total protein)	*p*‐value	Fold change
Aconitate	1.15 (0.69–1.28)	19.89 (14.55–28.50)	0.002	17.30
Alanine	83.12 (42.81–92.94)	365.26 (246.64–433.05)	0.002	4.39
Aspartate	451.73 (305.27–601.67)	454.46 (350.58–686.93)	ns	1.01
Citrate	5.67 (4.52–7.86)	14.86 (9.51–18.88)	0.004	2.62
Fructose 1,6‐BisP	209.44 (203.37–212.93)	5,619.52 (5,599.56–5,633.82)	0.002	26.83
Fructose‐6‐P	15.04 (12.44–19.31)	79.43 (42.78–123.19)	0.002	5.28
Fumarate	8.88 (6.73–11.62)	19.45 (15.47–21.85)	0.004	2.19
Glucose	281.14 (185.13–403.76)	6,510.54 (2,015.35–11,803.51)	0.010	23.16
Glucose‐6‐P	1.29 (1.17–1.92)	6.99 (5.96–13.50)	0.002	5.42
Glutamate	5,563.77 (3,232.82–7,326.42)	1,298.16 (1,134.68–2,988.12)	0.009	‐4.29
Glutamine	5.98 (4.45–7.79)	20.93 (16.56–29.99)	0.002	3.50
Isoleucine	43.00 (19.26–48.99)	81.00 (42.02–147.04)	ns	1.88
Lactate	1,584.41 (1,078.91–1,751.20)	6,062.51 (5,525.53–8,350.82)	0.004	3.83
Leucine	29.18 (15.52–36.19)	99.07 (57.98–190.48)	0.004	3.40
Pyruvate	13.74 (10.66–15.84)	87.25 (78.56–126.81)	0.004	6.35
Serine	4.51 (2.86–8.84)	35.06 (20.27–60.93)	0.004	7.77
Succinate	263.09 (185.92–309.85)	5,355.81 (4,742.19–5,721.65)	0.002	20.36
Valine	43.83 (25.40–49.95)	146.11 (84.52–208.71)	0.004	3.33

### 2.10 Statistical analysis

Each experiment was executed in triplicate. Results were analyzed by GraphPad Prism 6 statistical software and expressed as mean ± *SD*. Statistical analysis was performed using unpaired Student's *t* test and results with *p* value < .05 were considered statistically significant.

## 3 RESULTS

### 3.1 Establishment of hVW‐MSCs model from human femoral artery

**FIGURE 1:**
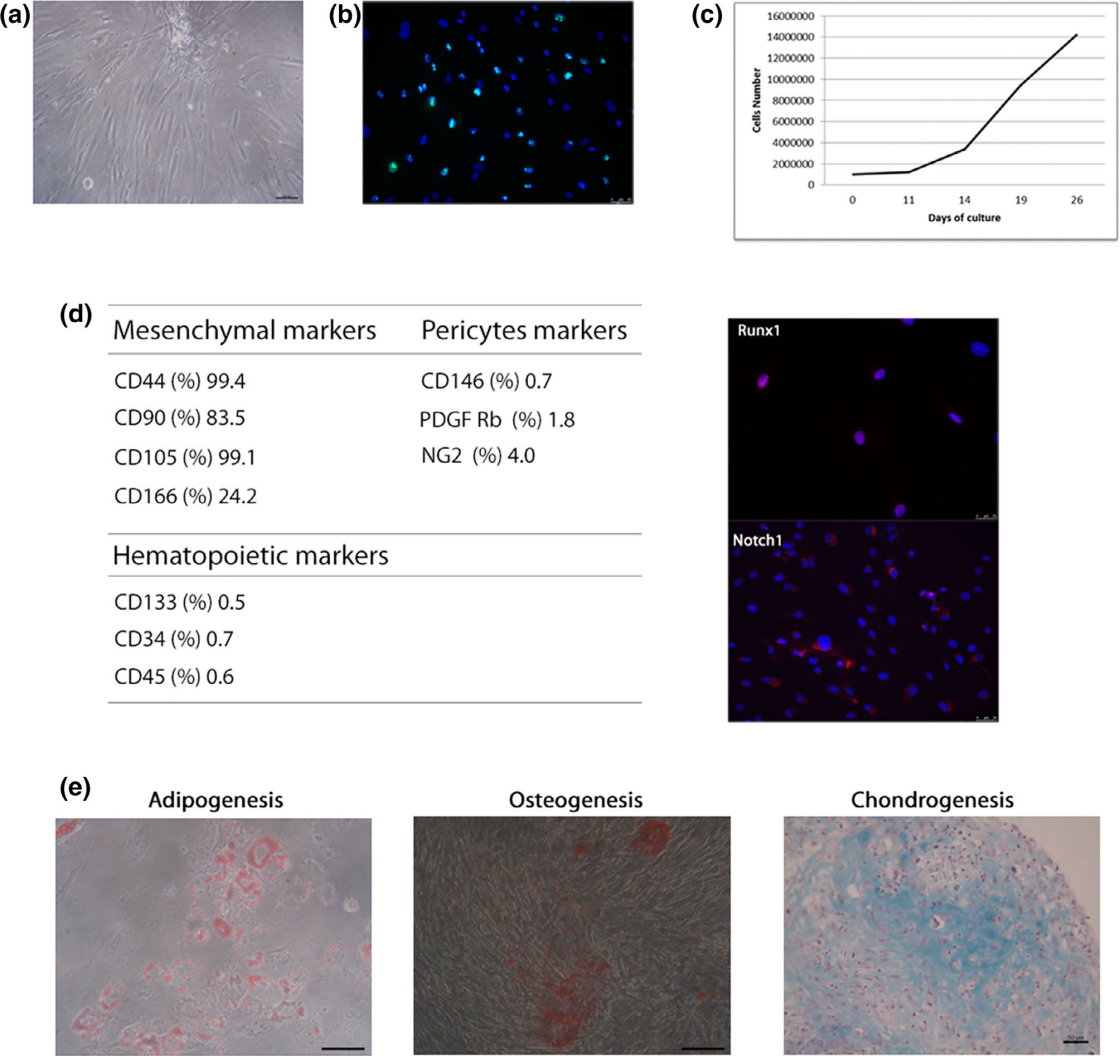
Isolation and characterization of hVW‐MSCs. Femoral hVW‐MSCs grown on 2D condition at passage 2 showed (a) a typical fibroblast‐like morphology with (b) high expression of the ki‐67 proliferation marker. (a) Scale bar: 100 μm; (b) Scale bar: 50 μm. (c) Growth kinetic of hVW‐MSCs; after 3 weeks of 2D culture, the cells were expanded 14‐fold resulting in 140 × 10^6^ cells. (d) Flow cytometry analysis for the mesenchymal (CD44, CD90, CD105, and CD166), pericytes (CD146, PDGF Rβ and NG2), and hematopoietic (CD133, CD34, and CD45) antigens (left) and immunofluorescence staining for stemness (Runx‐1 and Notch‐1) markers (right). (e) Mesengenic potential of hVW‐MSC under appropriate induction medium: in adipogenesis, lipidic drops were stained with Oil red O, scale bar: 100 μm; in osteogenesis, calcium deposits with Alizarin Red, scale bar: 100 μm; in chondrogenesis, proteoglycan‐rich extracellular matrix with Alcian Blue, scale bar: 50 μm

hVW‐MSCs, isolated from human femoral artery, are an adherent population characterized by typical spindle‐shape morphology and high proliferative capacity (Figure 1a–c). Both flow cytometry and immunofluorescence analysis showed a mesenchymal and stemness profile (CD44, CD90, CD105, CD166, Notch‐1 and Runx‐1), whereas the lack of hematopoietic (CD133, CD34 and CD45) and pericyte (CD146, PDGF Rβ and NG2) antigen expression was in agreement with the vascular wall residency (Figure 1d). The multilineage differentiation assays proved the hVW‐MSCs plasticity and their ability to differentiate into osteo/adipo/chondrogenic cell types (Figure 1e); overall, these results demonstrated the stemness nature of the cells recovered from femoral artery.

### 3.2 Generation of hVW‐MSCs spheroids, nature of cell aggregation, size and growth curve

HVW‐MSCs grown under nonadherent conditions using both low‐attachment and hanging drops techniques, spontaneously aggregated into spheroids. To indicate their perivascular niche origin, we refer to hVW‐MSC spheroids with the term of “vascular spheres.”

**FIGURE 2:**
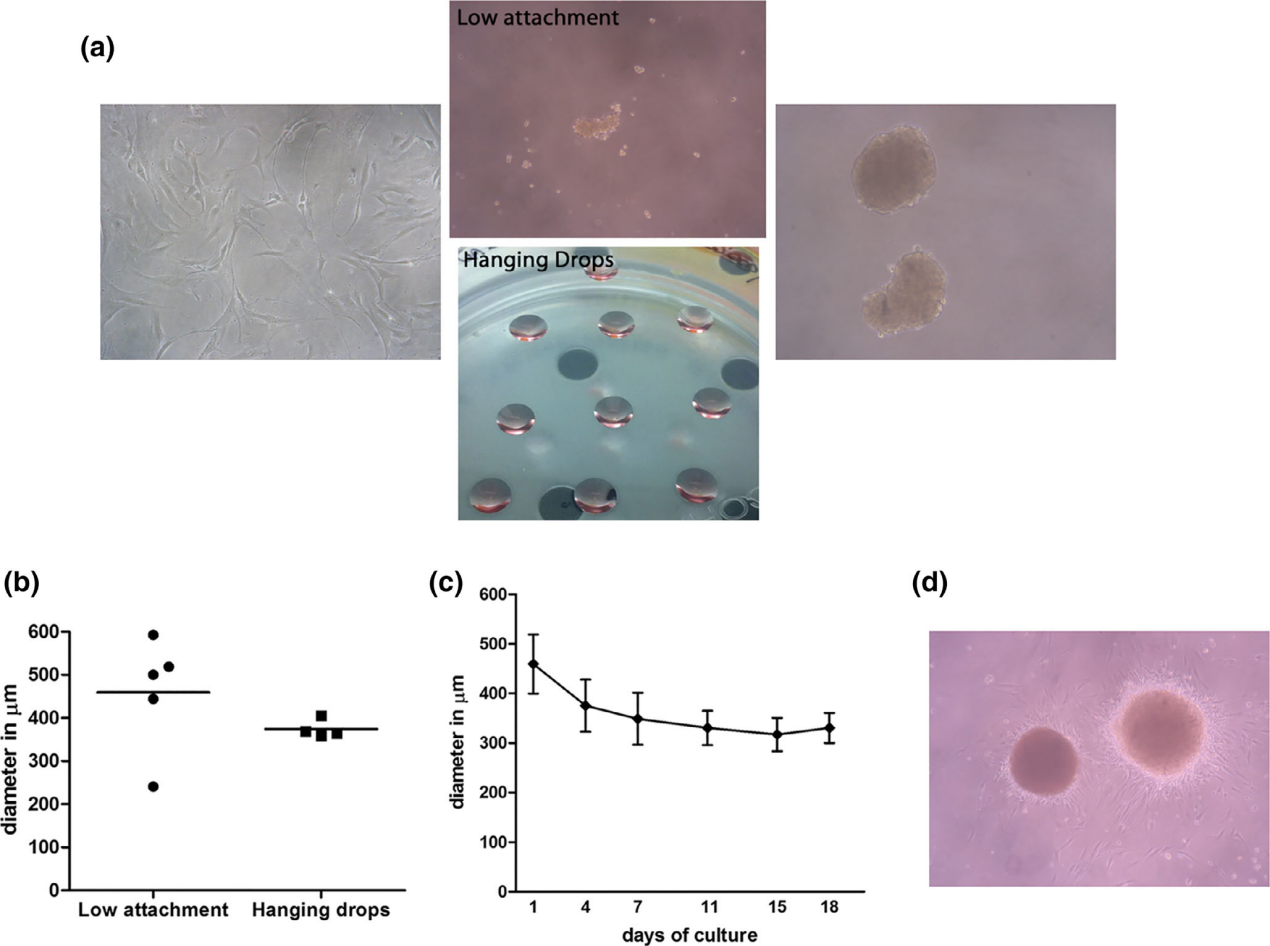
Formation of vascular‐spheres from hVW‐MSCs. (a) Confluent hVW‐MSCs grown as a 2D monolayer were detached and re‐suspended in basal culture medium to generate spheroids using low attachment plates or hanging drops methods. Representative image of spheroids after 3 days of culture. (b) Size distribution of vascular‐spheres derived from low attachment plate and hanging drops. (c) Growth curve of hVW‐MSC vascular‐spheres was followed for up 18 days of culture showing a progressive reduction in size (about 28%). Diameters were measured on five independent vascular‐spheres and express as average values. (d) Representative image of vascular‐spheres plated on 2D culture condition; after 1 day, the hVW‐MSC restored their mesenchymal morphology

After 3 days of culture, numerous floating 3D vascular‐spheres were observed using inverted LM (Figure 2a). Vascular‐spheres derived from ultra‐low attachment plates varied in size: small (about 240 μm in diameter) and large (about 600 μm in diameter); conversely, vascular‐spheres derived from hanging drops resulted more homogeneous in size, measuring an average of 350 μm (Figure 2b). To determine the cell number composition, single vascular‐spheres from low adherence plates were dissociated in individual cells and manually counted, revealing that vascular‐spheres were composed either by 5,973 or 1,686 cells respectively in large or small spheres. Furthermore, five independent vascular spheres were observed and photographed under the inverted LM for up 18 days; the growth curves revealed a size reduction of the spheres in a time‐dependent manner, losing about 28% of the original diameter (Figure 2c). The reversibility of the vascular‐sphere generation process was proved replating the vascular spheres in standard culture dishes; each vascular‐sphere adhered to the plastic surface and spread out giving origin to spindle mesenchymal cells (Figure 2d).

### 3.3 Immunophenotypic characterization and pluripotent gene expression of vascular‐sphere hVW‐MSC‐derived

**FIGURE 3:**
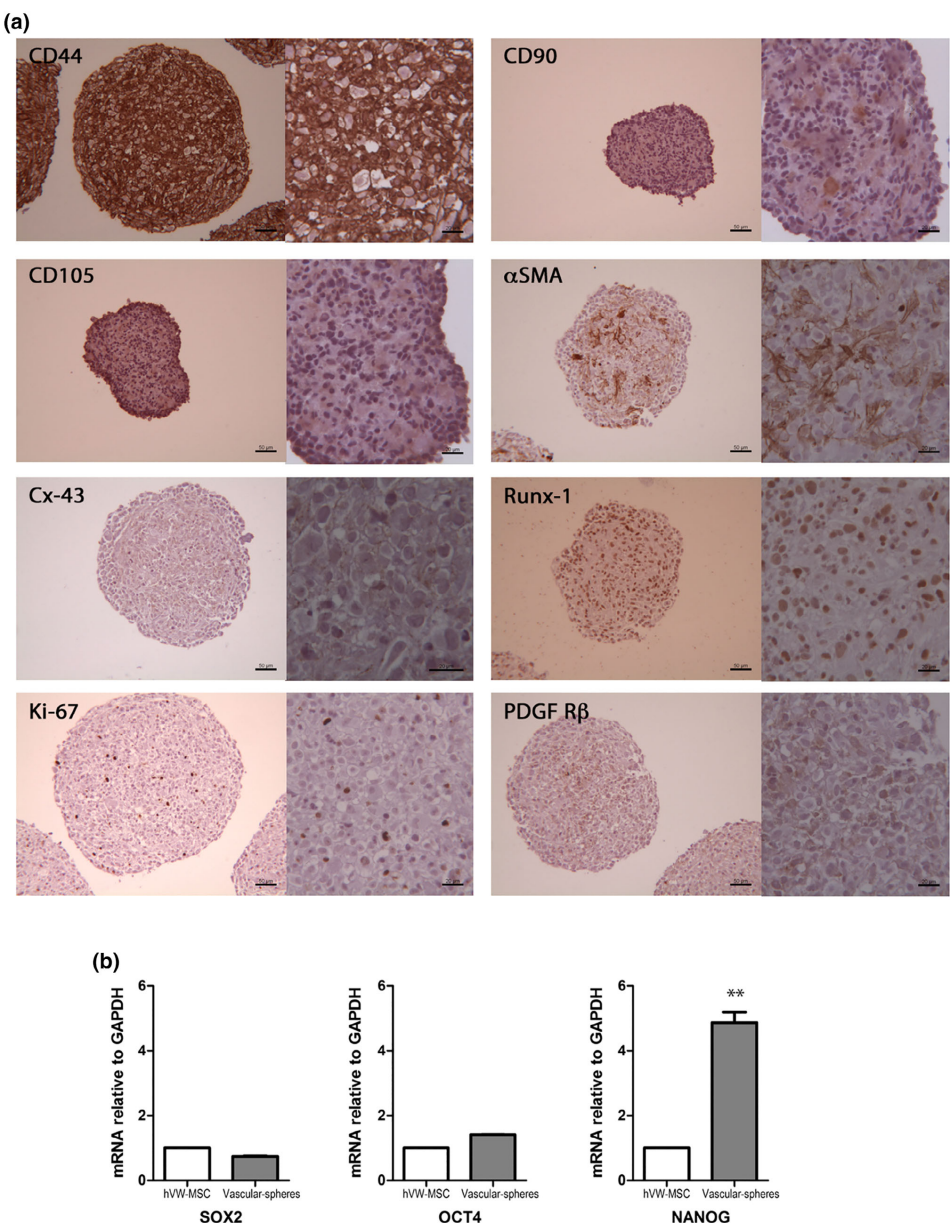
Characterization of vascular‐spheres. (a) hVW‐MSC vascular‐spheres were composed by cells intensely expressing CD44^+^; few CD90 and CD105 faintly positive cells were seen mostly in the internal layers; α‐SMA^+^ and Runx‐1^+^ cells localized in the inner layers; ki‐67^+^ proliferating cells in the outer layers. The PDGF Rβ^+^and Cx‐43^+^ expression was low and distributed in the entire vascular‐sphere. Scale bar for all images: 50 μm; all magnifications: 20 μm. (b) Real‐time PCR of stemness genes in vascular‐spheres. Results are expressed as fold relative to 2D. OCT4: octamer‐binding transcription factor‐4; SOX2 or SRY: sex determining region Y‐box 2. Statistical analysis was performed by unpaired Student's *t* test; **p < .01

The immunocytochemical analysis showed that cells composing the vascular spheres intensely and diffusely expressed the CD44 mesenchymal stem cell antigen; conversely, CD105 and CD90 expression was low. Runx‐1 positive cells were mainly seen in the core of the spheres; α‐SMA highlighted a delicate contractile network in the central portion of the sphere. PDGF‐Rβ and Cx‐43 were less expressed. Cycling cells positive to ki‐67 proliferation marker were seen in vascular spheres (Figure 3a); by counting proliferating cells over the total number of cells composing the vascular‐sphere revealed that more than 80% of cells were in G0 phase. Semiquantitative analysis by Real Time PCR revealed a significant fourfold increase of NANOG expression in vascular spheres compared to hVW‐MSC monolayer; a slight up‐regulation of Octamer‐binding transcription factor‐4 (OCT‐4) and a decrease of Sex determining region Y‐box 2 (SOX‐2) transcripts were observed in vascular spheres, but without statistical significance (Figure 3b).

### 3.4 Histological and ultrastructural organization of hVW‐MSCs inside vascular‐sphere

**FIGURE 4:**
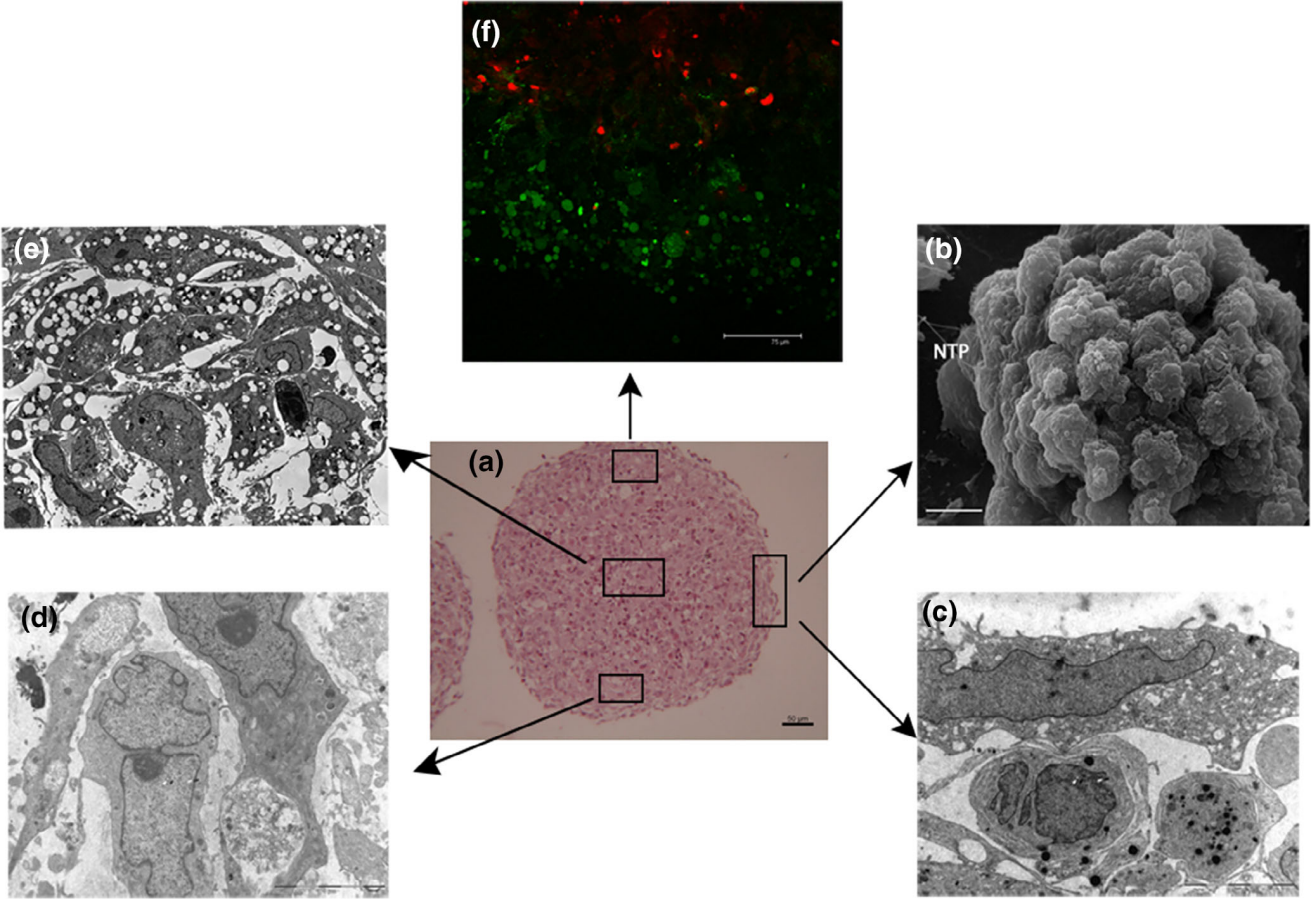
Histology and ultrastructure of vascular‐spheres. (a) Representative histological image of vascular‐spheres showing multiple hVW‐MSC layers surrounding a central core. Scale bar = 50 μm. (b) SEM analysis of vascular‐sphere surface showing outer cells with smooth and bullous surfaces and nanotubular projections (NTPs). Scale bar: 10 μm. (c) TEM of outer cells confirms the smooth surface and poor organelle content. (d) Subsurface cells show large euchromatic nuclei with nucleoli, small mitochondria and glycogen deposits. (e) Loose cell aggregation and presence of apoptotic cells are features of the internal portion of large vascular‐spheres. (c–e) Scale bars = 5 μm. (f) LIVE/DEATH assay revealed that the viable cells (green) were principally disposed in outer layers while the death cells (red) occupied the central core of the vascular‐spheres. Scale bar = 75 μm

**FIGURE 5:**
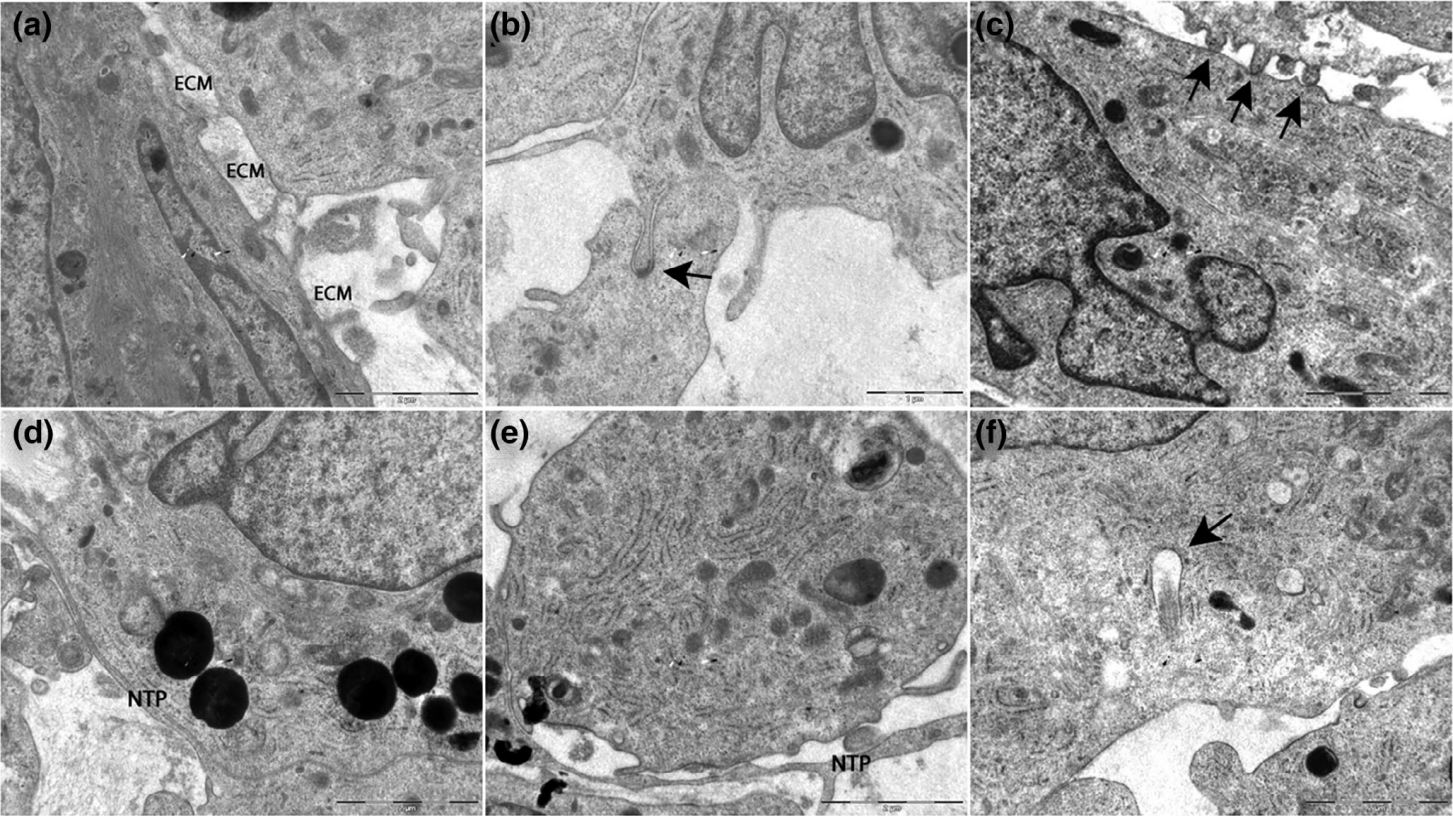
Mesenchymal cell identity and quiescent status of vascular‐spheres. (a) Focal extracellular matrix (ECM) deposition in the extracellular space. (a) scale bar = 2 μm. (b,c) Intercellular junctions of mesenchymal type: the arrows indicate a subplasmalemmal linear densities in (b) and club‐shaped contacts in (c). (b) Scale bar = 2 μm; (c) scale bar = 1 μm. Linear (d) and branched (e) nanotubular projections (NTP). (f) Primary cilium (arrow). (d–f) Scale bars = 2 μm

Histologically, several layers of spindle cells enclosing rounded cells characterize vascular‐spheres (Figure 4a). SEM analysis documented that the surface of outer cells had a smooth, bullous membrane emitting thin and long projections touching target cells (Figure 4b); no microvilli were seen. By TEM, the vascular‐spheres were composed by undifferentiated spindle cells with irregularly shaped nuclei containing dispersed chromatin and prominent nucleoli; the cytoplasm contained small, dark mitochondria, well‐developed rough endoplasmic reticulum (rER) cisternae and glycogen deposits (Figure 4c,d). In the central core, hVW‐MSCs were rounded and loosely arranged (Figure 4e); consistent with their mesenchymal nature, they delimit an extracellular space into which matrix material was seen focally (Figure 5a); likewise, cell‐to‐cell contacts of mesenchymal nature, such as subplasmalemmal paired densities and club‐shaped interdigitations (Figure 5b,c), were seen. Live cells mixed with necrotic and apoptotic cells (Figure 4d,e) were observed in the central area; these results were confirmed by LIVE/DEATH assay. In detail, the dead cells (intensely stained in red) were localized in the core of the vascular‐spheres while living cells (marked in green) were located principally in the outer layers of vascular‐spheres (Figure 4f). In addition to the long, thin projections already seen in the surface cells, TEM revealed an articulate cell to cell communication network composed of multivesicular bodies enclosing exosomes and long, thin linear or branched cytoplasmic extensions with bifurcations (Figure 5d,e). Moreover, solitary flagella, that is, primary cilia (Figure 5f), were seen at a frequency of 1–2 cilia for spheroid section, composed of a mean of 100 cells.

### 3.5 Metabolomic analysis of vascular‐sphere and their parental hVW‐MSC cells

**FIGURE 6:**
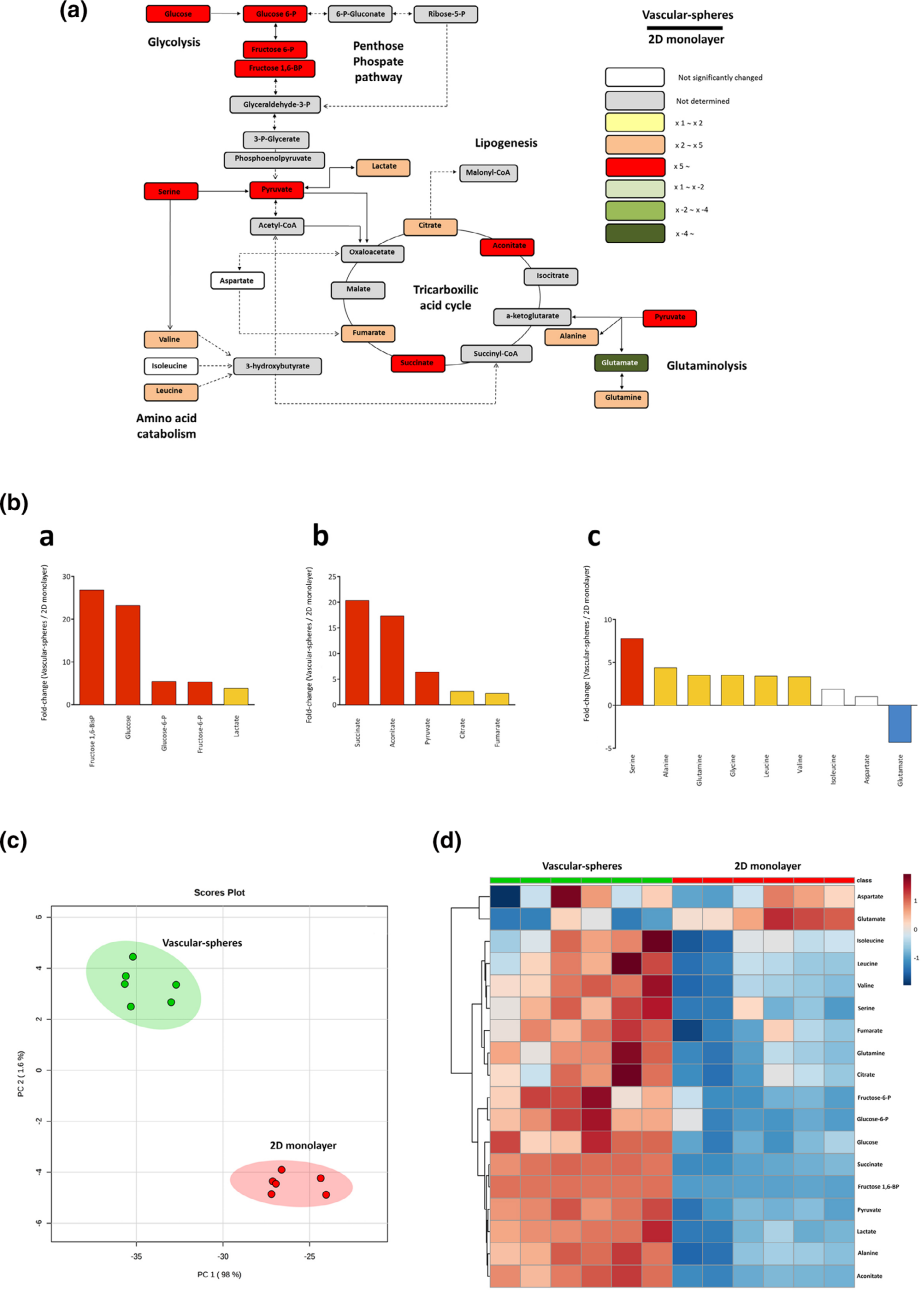
Metabolomic analysis of vascular‐spheres. (a) Schematic representation of energy metabolism. Metabolites are graphically represented comparing 3D vascular‐spheres versus 2D monolayer culture hVW‐MSC. Statistically significant differences are considered when *p*‐value > .05. (b) Fold‐change values of metabolites segregated by metabolic pathways: glycolysis (a), tricarboxylic acid cycle (b) and amino acid metabolism (c). (c) Principal component analysis plot showing differences between hVW‐MSC vascular‐sphere and their parental monolayer cells. (d) Heatmap representation showing a different pattern in vascular‐spheres and their parental monolayer cells. Rows: metabolites. Columns: individual samples. Blue color (low values); red color (high values)

The metabolomics analysis mirrored a differential metabolism between the 2D and the 3D growth conditions. We analyzed the third passage of hVW‐MSC cultured both on 2D monolayer and 3D vascular‐spheres. Metabolite concentrations, p‐values and fold‐change are shown in Table 3. As seen in Figure 6a, most of the detected metabolites had fold‐changes over value 2. Fold‐change values are displayed in Figure 6b. Metabolites of glycolytic pathway (Figure 6b (a) (glucose, glucose‐6‐phosphate, fructose‐6‐phosphate and fructose‐1,6‐bisphosphate) were accumulated in spheroids. Moreover, citrate, aconitate, fumarate, succinate (metabolites of tricarboxylic acid cycle, Figure 6b (b), valine and leucine (metabolites of amino acid catabolism, Figure 6b (c) had high fold‐change values, indicating that its concentration was higher in vascular‐spheres compared to monolayer hVW‐MSC. If displayed in a principal component analysis (PCA) plot (Figure 6c), vascular‐spheres and monolayer hVW‐MSC showed different behavior. They can be completely differentiated by using this statistical analysis. The heatmap (Figure 6d) showed a clear pattern of differentiation between cells: most of the metabolites were high (red) in vascular‐spheres if compared with monolayer hVW‐MSC.

## 4 DISCUSSION

The present study was aimed at characterizing vascular‐spheres obtained from 3D culture of hVW‐MSCs, combining the reliability of primary cultures with the accuracy of the 3D environment, which mimics the stemness niche.

Femoral artery hVW‐MSCs were able to rapidly and spontaneously aggregate into vascular‐spheres, composed of CD44^+^, CD105^low^, CD90^low^ mesenchymal cells. Accordingly, a CD44^+^ multipotent stem cell population with multilineage mesodermal plasticity, including smooth muscle cells and pericytes, was identified in the adventitia of adult fresh human internal thoracic artery (Klein et al., 2011). Further, we found a significant up‐regulation of NANOG in vascular‐spheres, hypothesizing that 3D culture enhances stemness and pluripotency features of hVW‐MSCs. This condition is also supported by the ability of hVW‐MSCs to survive anoikis, a form of programmed cell death that occurs when anchorage dependent cells, like mesenchymal cells, grow under non‐adherent conditions without extracellular matrix support. Indeed, the resistance to lethal or sublethal stresses is suggestive of an elevated hierarchical status (Ciavarella et al., 2015), therefore it could be speculated that hVW‐MSCs within spheres may be ancestor MSCs.

An interesting question concerns the methods of spheroid formation, considered to derive from tight cell aggregates (Rajcevic et al., 2014); theoretically, vascular‐spheres should originate from cell division or cell adhesion. We observed only few and peripheral cells positive to ki‐67, a well‐known proliferation marker, in vascular spheres; therefore, we hypothesized that more than 80% of hVW‐MSC composing the vascular‐spheres were in G0 phase, in contrast with the proliferation rate seen in 2D conditions, and that vascular‐spheres derive from adhesion of anchorage independent mesenchymal cells. The low ki‐67 expression also indicates a quiescent status, coherent with the niche environment. At this regard, TEM shows cells exhibiting a primary cilium: this solitary flagella arising at the surface of non‐proliferating cells, is involved in the control of cell quiescence, and it was never described in normal vascular cells, even though Haust found it in smooth muscle cells in human atherosclerotic lesions (Haust, 1987). A recent study demonstrated increased proliferation in MSCs following siRNA knockdown of the ciliary proteins IFT_172_ and KIF_3_A, and the down‐regulation of OCT‐4, SOX‐2 and NANOG, confirming that primary cilium is crucial to MSC proliferation and stemness (Ma et al., 2020). Further, primary cilia regulate many signaling pathways associated with cell differentiation and lineage specification, suggesting their potential application as novel therapeutic target (Bodle & Loboa, 2016).

In addition, we observed a time‐dependent reduction of vascular‐spheres, thus indicating that cell loss prevailed over cell proliferation. Possible explanations for the high rate of quiescent cells seen in mesenchymal spheroids could be cell selection through anchorage impediment, aggregation modality, remodeling of cell shape, gravity or other physical force influence, floating, low nutrient support or oxygen availability.

Another interesting finding of this study regards how anchorage‐independent mesenchymal cells aggregate to form vascular‐spheres. Since these cells are predominantly quiescent, it could be that physical forces trigger their aggregation; this may also explain why variously sized spheres were generated in relation to the culture method employed. In the ultra‐low attachment plates, Brownian motion and interfacial tension facilitate cell aggregation as well as fusion of individual MSCs into multi‐cell aggregates thanks to unbalanced intermolecular forces between medium and spheres. Accordingly, when the vascular‐spheres are immersed in the medium, they undergo random motion and a large number of collisions; after that, single spheres come together and merge forming larger spheres to reduce the area subjected to interfacial tension. In contrast, in the hanging drop method, the aggregation of mesenchymal cells was promoted by the gravity force exclusively; consequently, individual spheres were generated for each drop and all spheres obtained remained similar in size being the result of aggregation and not proliferation. The effect of physical forces on vascular sphere generation is supported by the following aspects: (i) the presence of smooth surfaces without microvilli on the luminal MSCs as documented by SEM, a strategy to minimize the surface volume exposed to external forces, (ii) the loose cell aggregation as revealed by TEM, in contrast with previous beliefs that spheres are composed by tightly adherent cells, (iii) the immunohistochemical topographical expression of α‐actin that reflects cell attempts to maintain multi‐cell cohesion counteracting external physical forces.

Histology and ultrastructural analysis revealed that hVW‐MSC cells had a loose arrangement coherent with their mesenchymal nature; they were joined by subplasmalemmal linear densities (mesenchymal‐type junctions) and delivered extracellular matrix focally. Runx‐1, a key transcription factor for MSC proliferation was weakly expressed in the core; Connexin‐43 was faint and scattered throughout the sphere. The weak expression of PDGF‐Rβ and the lack of CD146 support the hypothesis that not all of the mesenchymal cells may be related to pericytes as recently suggested by de Souza, Malta, Kashima Haddad, and Covas (2016). Accordingly to this view, when vascular‐spheres were plated in 2D standard condition, hVW‐MSC cells spread out from 3D spheres restoring their typical spindle shape morphology. Thus, sphere formation is a reversible and momentary ability of a subtype of MSC with particular resistance to anchorage‐impairment stress.

As previously reported (Bartosh et al., 2010), also vascular‐spheres are histologically composed by two distinct areas with a peripheral zone of multilayered spindle hVW‐MSC cells and a core zone of rounded cells. Previous studies reported that the central core of the vascular‐spheres contained mostly dead cells as a consequence of low oxygen availability, the entity of dead cells correlating with spheroids size (more than 200–250 μm in diameter) (Curcio et al., 2007). Also our study showed a mixture of live and dead cells; confocal fluorescent microscopy of 300–350 μm vascular‐spheres localized dead cells in the inner core. TEM provided evidence that a significant proportion of dead cells were in apoptosis, suggesting that poor access to nutrients or oxygen are not exclusive determinants of core necrosis; in fact, SEM and TEM showed multiple 100 nm sized nanotubular projections as those described in a previous study by our group (Valente, Rossi, Resta, & Pasquinelli, 2015). These thin membranous channels represent a novel communication system of exchanging molecules and cytoplasmic organelles among cells also placed at a significant distance from the point of origin of the projection itself; in addition to being a structural requirement for the exchange of biological information (Simons & Raposo, 2009), nanotubular projections can constitute a system for feeding cells located in an unfavorable positions or eliminating waste products. In our view, the presence of core apoptosis may reflect the effect due to the absence of nutrient and the alteration of the catabolic process.

Further, metabolic analysis supports the quiescent status of hVW‐MSCs composing the vascular‐spheres. The regulation of central metabolic pathways is considered an important modulator of stem cell quiescence and pluripotency. By the inhibition or enhancement of key processes, stem cells regulate energy production using nutrient‐sensing pathways (Ochocki & Simon, 2013). The accumulation of metabolites from glycolysis, tricarboxylic acid cycle and some related to amino acid catabolism may indicate a slower metabolism within spheroids. To sustain cell functions, higher amounts of energy are necessary, while there is a lower need for anabolic precursors. In this case, all metabolic pathways are redirected to complement tricarboxylic acid cycle, to obtain energy for cell survival. This characteristic is supported by other metabolomic experiments, in which citrate, glutamate and malate were decreased in embryonic stem cells and induced pluripotent stem cells when compared to somatic cells (Vacanti & Metallo, 2013). Results in embryonic stem cells showed glycolysis is one of the most active components of their metabolism, as they produce more lactate and consume more glucose than differentiated cells (Ochocki & Simon, 2013; Zhang et al., 2011).

Spheroids showed high values of glutamine, although glutamate values were lower. This can be explained because, apart from glucose, glutamine can be a substrate for energy production. Glutamine provides a source of nitrogen to proliferating cells for amino acid synthesis. Though, if cells are not proliferating, glutamine is not being consumed, and then there is no production of glutamate (Li, Zhang, Zhao, Ma, & Chen, 2016; Vacanti & Metallo, 2013). To maintain the metabolite flux in the tricarboxylic acid cycle, anaplerotic pathways such as glutaminolysis are often necessary. In this case, glutamine is an important substrate (Vacanti & Metallo, 2013).

## 5 CONCLUSION

This study reports the characteristics of hVW‐MSCs derived from human femoral arteries when cultured as vascular‐spheres under non‐adherent conditions. This 3D model is a surrogate of the physiological vascular niche microenvironment in which the hVW‐MSCs in vivo reside. The culture methods employed in the generation of vascular‐sphere reversibly select a CD44^+^, CD105^low^, CD90^low^ vascular mesenchymal cell population that is highly resistant to anchorage‐impairment stress and, when driven by physical forces, acquires the ability to aggregate rearranging the contractile cytoskeleton without losing the mesenchymal immunophenotypic and stemness markers. Followed up 18 days, the growth of vascular‐spheres remain stationary; along with ki‐67 staining and metabolomic experiments, these findings indicate that vascular‐spheres are mainly composed of quiescent hVW‐MSCs. Vascular‐spheres of hVW‐MSC should provide an improved understanding of the vascular stem cell biology with an estimated impact on organogenesis, tissue engineering and regenerative medicine for future clinical applications.

## CONFLICT OF INTEREST

The authors declare that there are no conflicts of interest regarding the publication of this article.
